# Genetic networks in the mouse retina: *Growth Associated Protein 43* and *Phosphatase Tensin Homolog* network

**Published:** 2011-05-25

**Authors:** Natalie E. Freeman, Justin P. Templeton, William E. Orr, Lu Lu, Robert W. Williams, Eldon E. Geisert

**Affiliations:** 1 Department of Ophthalmology and Center for Vision Research, Memphis, TN; 2 Department of Anatomy and Neurobiology and Center for Integrative and Translational Genomics, University of Tennessee Health Science Center, Memphis, TN

## Abstract

**Purpose:**

The present study examines the structure and covariance of endogenous variation in gene expression across the recently expanded family of C57BL/6J (B) X DBA/2J (D) Recombinant Inbred (BXD RI) strains of mice. This work is accompanied by a highly interactive database that can be used to generate and test specific hypotheses. For example, we define the genetic network regulating growth associated protein 43 (*Gap43*) and phosphatase tensin homolog (*Pten*).

**Methods:**

The Hamilton Eye Institute (HEI) Retina Database within GeneNetwork features the data analysis of 346 Illumina Sentrix BeadChip Arrays (mouse whole genome-6 version 2). Eighty strains of mice are presented, including 75 BXD RI strains, the parental strains (C57BL/6J and DBA/2J), the reciprocal crosses, and the BALB/cByJ mice. Independent biologic samples for at least two animals from each gender were obtained with a narrow age range (48 to 118 days). Total RNA was prepared followed by the production of biotinylated cRNAs, which were pipetted into the Mouse WG-6V2 arrays. The data was globally normalized with rank invariant and stabilization (2z+8).

**Results:**

The HEI Retina Database is located on the GeneNetwork website. The database was used to extract unique transcriptome signatures for specific cell types in the retina (retinal pigment epithelial, amacrine, and retinal ganglion cells). Two genes associated with axonal outgrowth (*Gap43* and *Pten*) were used to display the power of this new retina database. Bioinformatic tools located within GeneNetwork in conjunction with the HEI Retina Database were used to identify the unique signature Quantitative Trait Loci (QTLs) for *Gap43* and *Pten* on chromosomes 1, 2, 12, 15, 16, and 19. *Gap43* and *Pten* possess networks that are similar to ganglion cell networks that may be associated with axonal growth in the mouse retina. This network involves high correlations of transcription factors (SRY sex determining region Y-box 2 [*Sox2*], paired box gene 6 [*Pax6*], and neurogenic differentiation 1 [*Neurod1*]), and genes involved in DNA binding (proliferating cell nuclear antigen [*Pcna*] and zinc finger, BED-type containing 4 [*Zbed4*]), as well as an inhibitor of DNA binding (inhibitor of DNA binding 2, dominant negative helix–loop–helix protein [*Id2*]). Furthermore, we identified the potential upstream modifiers on chromosome 2 (teashirt zinc finger homeobox 2 [*Tshz2*], RNA export 1 homolog [*Rae1*] and basic helix–loop–helix domain contatining, class B4 [*Bhlhb4*]) on chromosome 15 (RAB, member of RAS oncogene family-like 2a [*Rabl2a*], phosphomannomutase 1 [*Pmm1*], copine VIII [*Cpne8*], and fibulin 1 [*Fbln1*]).

**Conclusions:**

The endogenous variation in mRNA levels among BXD RI strains can be used to explore and test expression networks underlying variation in retina structure, function, and disease susceptibility. The *Gap43* and *Pten* network highlights the covariance of gene expression and forms a molecular network associated with axonal outgrowth in the adult retina.

## Introduction

A key challenge in ophthalmology is defining genetic and molecular mechanisms that underlie retinal disease. Recent advances in genomics offer great opportunities to begin to unravel complex genetic networks that often contribute significantly to retinal diseases such as glaucoma [1,2], macular degeneration [3,4], and retinopathies [5-7]. Studies in humans are of course the main drivers of this research program, but it is often significant to have well defined genetic models for detailed mechanistic and molecular analysis. One novel and powerful approach that we have exploited combines elements of systems biology and complex trait analysis by using a genetic reference panel that consists of more than 75 C57BL/6J (B) X DBA/2J (D) Recombinant Inbred (BXD RI) strains of mice [8-14].

Collectively this large family provides a powerful system to integrate baseline phenotypes of nominally wild-type strains, a) with heritable risk of disease (for example, many of the BXD strains develop a severe form of glaucoma) [15-18]; b) with natural variation in expression and other traits, such as cell population sizes [15,16,19]; c) with matched studies on effects of interventions on disease progression; and finally d) with the ability to identify some of the causal gene variants segregating within this family that contribute to ocular disease [12,13,20].

**Figure 1:**
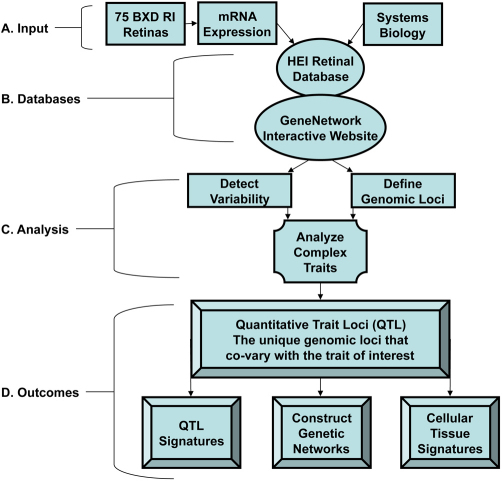
The Hamilton Eye Institute (HEI) Retina Database and GeneNetwork can reveal the covariance in gene expression that establishes genetic networks in the retina. **A**: Input: A systems biology approach and the microarray data from 75 BXD RI mouse retinas feed into the HEI Retina Database. **B**: Databases: The HEI Retina Database links to the interactive GeneNetwork Database website. **C**: Analysis: The analytical tools within GeneNetwork are used to interrogate the data set and detect the co-variance among sets of genes that map to a common genetic locus and collectively control a complex trait. **D**: Outcomes: Identify the unique genomic loci that co-vary with the trait of interest, which lends to the discovery of quantitative trait loci (QTL), cellular, and tissue signatures. These signatures allow the construction of schema for genetic networks.

The second component—the systems biology approach—takes advantage of high throughput expression profiling of the entire family of strains. The expression data from 80 strains and both sexes would be overwhelming without special analytic tools, and for that reason we have integrated the entire data set in GeneNetwork for rapid online analysis. In Figure 1, we illustrate the process of exploiting natural perturbations of gene expression to define specific molecular networks in retina. We provide one example that highlights a network associated with axonal outgrowth in the adult mouse retina. This network is defined in part by high natural covariance among transcription factors: SRY (sex determining region Y)-box 2 [*Sox2*] and paired box gene 6 [*Pax6*], neurogenic differentiation 1 [*Neurod1*]), genes involved in axonal outgrowth (growth associated protein 43 [*Gap43*] and phosphatase tensin homolog [*Pten*]), genes involved in DNA binding (proliferating cell nuclear antigen [*Pcna*] and zinc finger, BED-type containing 4 [*Zbed4*]), and an inhibitor of DNA binding [inhibitor of DNA binding 2, dominant negative helix–loop–helix protein [*Id2*]). The use of a systems genetics approach that exploits large expression data sets for entire families of strains with online resources provides a powerful and practical way to study and test the genetics of retinal function and disease susceptibility.

## Methods

### Animals: Strains, sex, and age

Benjamin A. Taylor at The Jackson Laboratory (Bar Harbor, Maine) generated the BXD RI mouse strains 1 through 42; BXD RI 1 through BXD RI 32 strains were generated starting in the late 1970s [21] and the BXD RI 33 through BXD RI 42 were bred in the 1990s [22]. BXD RI strains 43 through 103 were generated in the late 1990s and early 2000s using advanced intercross progeny by Lu Lu, Jeremy Peirce, Lee M. Silver, and Robert W. Williams [23]. Collectively, 80 strains of mice are represented within the Hamilton Eye Institute (HEI) Retina Database, including 75 BXD RI strains (n=307), C57BL/6J (n=4), DBA/2J (n=4), the progeny of reciprocal F1 crosses of C57BL/6J (n=4), and DBA/2J (n=4), as well as BALB/cByJ (n=4) mice. All animals used within this study were purchased from The Jackson Laboratory or were obtained from the breeding colonies of Drs. Robert Williams and Lu Lu at the University of Tennessee Health Science Center (UTHSC, Memphis, TN). Our goal was to collect data for independent biologic samples from both male and female mice for all the available BXD RI strains. All of the animals were young adults between 48 and 118 days of age (see Appendix 1). All mice were housed in standard cages with water and chow available ad libitum. The housing room was maintained on a reversed 12 h:12 h (6:00 AM–6:00 PM) light–dark cycle. All protocols used in this study were approved by the Animal Care and Use Committee of the UTHSC and were in accordance with the Institute for Laboratory Animal Research and with the Association of Research in Vision and Ophthalmology (ARVO) Statement for the Use of Animals in Ophthalmic and Vision Research.

### Replication and samples within the database

Collectively, our data represent sampling of males (n=162) and females (n=164) from 80 mouse strains (total n=326), without within-strain-by-sex replication. Our goal was to obtain data for independent biologic samples including at least two animals from each sex for all BXD RI mouse strains. We were able to obtain four animals from a total of 64 strains of BXD RI mice, two male (M) and two female (F), totaling 256 retina samples. In 13 of the 80 BXD RI strains, we were able to obtain greater than four samples per strain: BXD RI 1 (n=4 F, n=2 M), 32 (n=3 F, n=2 M), 38 (n=4 F, n=2 M), 40 (n=2 F, n=3 M), 42 (n=3 F, n=2 M), 49 (n=2 F, n=3 M), 60 (n=3 F, n=2 M), 62 (n=2 F, n=3 M), 68 (n=2 F, n=5 M), 70 (n=2 F, n=3 M), 73 (n=4 F, n=2 M), 83 (n=2 F, n=3 M), and 103 (n=4 F, n=3 M). Strict quality control (defined below) and the lack of availability resulted in 16 strains in which we were unable to collect two samples for one or both genders: BXD RI 5 (n=4 F, n=0 M), 16 (n=1 F, n=2 M), 31 (n=2 F, n=1 M), 39 (n=2 F, n=1 M), 44 (n=1 F, n=1 M), 45 (n=4 F, n=1 M), 51 (n=1 F, n=2 M), 55 (n=1 F, n=2 M), 56 (n=1 F, n=3 M), 66 (n=1 F, n=2 M), 71 (n=1 F, n=2 M), 80 (n=1 F, n=2 M), 81 (n=2 F, n=1 M), 89 (n=1 F, n=3 M), 92 (n=1 M; species extinct), and 102 (n=2 F, n=1 M). For clarification regarding the BXD RI 24 strain, there are two strains of BXD RI 24: BXD RI 24A and 24. BXD RI 24A was cryorecovered in 2004 from a stock of embryos (1988; generation 80 [F80]), and this strain does not exhibit retinal degeneration (The Jackson Laboratory strain identification is BXD24/TyJ). The BXD RI 24 strain acquired a spontaneous mutation in the centrosomal protein 290 (*Cep290*) at the retinal degeneration16 allele (*rd16*), which resulted in retinal degeneration [24]. The Jackson Laboratory now denotes this strain as BXD24b/TyJ. We have used BXD RI 24 to denote this strain in our database since this is the terminology used throughout all of the BXD RI strain set databases.

### Tissue and sample processing

All animals were sacrificed by rapid cervical dislocation, and the retinas were removed immediately. The retinas were harvested during the light cycle within the time frame 10:30 AM to 4:00 PM. Two retinas per mouse were immersed in RNALater (Applied Biosystems, Foster City, CA) and stored (4 °C) in a single tube overnight. The next day the retinas were transferred to the freezer (−20 °C) for at least 24 h and then placed in long-term storage (−80 °C). Total RNA was prepared from the retinal tissue with RNA-Stat-60 as described by the manufacturer (Tel-Test, Friendswood, TX). RNA-Stat-60 (Tel-Test) is composed of phenol and guanidinium thiocyanate in a mono-phase solution, which aids in the homogenization of the tissue. The addition of chloroform (Sigma Aldrich, St. Louis, MO) creates a biphasic solution composed of an aqueous and organic phase. The total RNA remains in the aqueous phase while the DNA and protein are extracted into an organic inter-phase. The total RNA is precipitated from the aqueous phase with isopropanol (Sigma Aldrich), washed with ethanol (Decon Laboratories, Inc., King of Prussia, PA) and the RNA is resuspended in nuclease free water (Applied Biosystems, Foster City, CA). The concentration of the RNA solution was determined by measuring the absorbance at 260 nm and 280 nm with the NanoDrop 1000A spectrophotometer (NanoDrop Technologies, Wilmington, DE). The quality and purity of RNA was assessed using an Agilent Bioanalyzer 2100 system (Santa Clara, CA; housed at UTHSC Molecular Research Center, Memphis, TN) determining the relative quantities of 18S and 28S RNA as well as the RNA integrity number (RIN). The RIN classifies the total RNA indicating the presence of contamination and evidence of RNA degradation based upon a numerical value of 1–10. The ideal standard for frozen tissue is an RIN value greater than 7, but for our studies we chose a value of 8 or greater.

### Biotinylation of cRNA, hybridization, and samples

Total RNA (150 ng) was processed with the Illumina TotalPrep RNA Amplification Kit (Applied Biosystems) to produce biotinylated complementary-RNAs (cRNAs). This procedure included the reverse transcription of RNA to synthesize the first strand complementary-DNA (cDNA), second strand cDNA synthesis, cDNA purification, in vitro transcription to synthesize cRNA, cRNA amplification, and purification. The concentration of the cRNA solution was determined by measuring the absorbance at 260 nm/280 nm using the NanoDrop 1000A spectrophotometer. The biotinylated cRNAs (1.5 µg/sample) were hybridized to the Illumina Sentrix® Mouse Whole Genome-6 version 2.0 arrays (Illumina, San Diego, CA) for 19.5 h at 58 °C (Illumina, San Diego, CA).

To avoid false genetic differences that lead to the reduction in the number of false cis-acting effects and the inflation of false trans-acting effects, samples from each strain were always pipetted onto different slides. Additionally, the samples were loaded at random locations within the chip and no two samples from a single strain were processed in a single chip.

### Illumina mouse genome arrays, annotation, and statistical analysis

The HEI Retina Database contains the data analysis of 346 Illumina Sentrix® Mouse Whole Genome-6 version 2.0 arrays (Illumina, San Diego, CA). The Mouse Whole Genome-6 version 2.0 BeadChips contain approximately 45,000 probes per array based on RefSeq Release 22 and are supplemented with Mouse Exonic Evidence Based Oligonucleotide (MEEBO) and content from the Functional Annotation of Mouse for the RIKEN full-length cDNA clone (RIKEN) [25,26]. The Illumina bead array confocal scanner and Illumina BeadStudio version 3.3.7 (Illumina) were used to scan the BeadChips and monitor the hybridization signals. The quality of the hybridization and overall processing of the BeadChips were monitored by visual inspection of both the internal quality control checklist and raw scanned data.

A quality control analysis was performed within the Illumina BeadStudio software to eliminate the systematic variation of nonbiologic origin within the raw microarray data using a normalization algorithm. A summary of the normalization can be found in the Illumina User Guide Review (Genome Studio, Gene Expression Module version 1.0 User Guide). Rank invariant normalization within the BeadStudio software was used to calculate the data. In addition, Minimum Information About a Microarray Experiment (MIAME) standards [27] were used for all microarray data. Once this data was collected, a Pearson correlation matrix was created between all the samples. The data was globally normalized in a four step process: 1) Compute the log base two of each raw signal value. 2) Calculate the mean and standard deviation of each mouse WG-6 v2.0 array. 3) Normalize each array using Equation 2z+8. The result is to produce arrays that have a mean of 8 and a standard deviation of 2. 4) Compute the mean of the values for the set of microarrays for each strain. Technical replicates were averaged before computing the mean for independent biologic samples. These data do not have a special correction for batch effects. The values in expression range from 6.25 to 18.08. The lowest level of expression is 6.25 for Illumina (ILMN)_2747167 (Rho GTPase activating protein 11A [*Arhgap11a*]). The highest level of expression is 18.08 for ILMN_2516699 (ubiquitin B [*Ubb*]).

## Results

### Genetic control of transcription in the mouse retina

**Figure 2:**
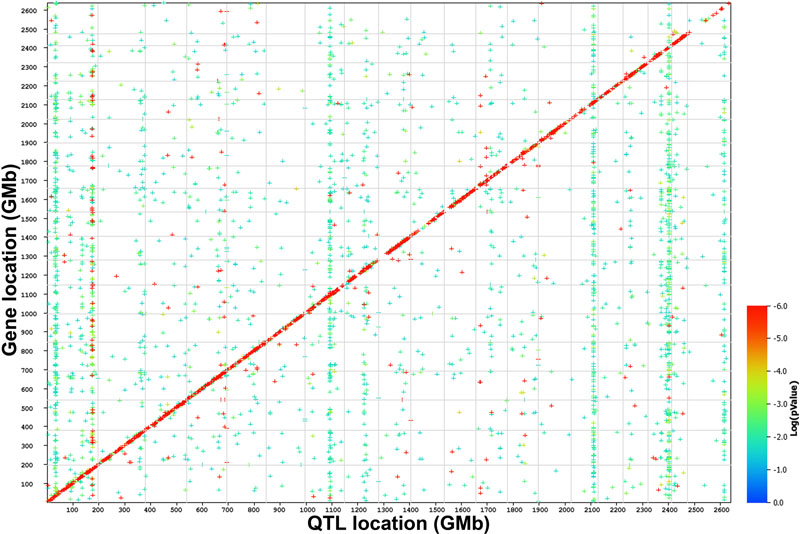
A genome-wide graph displaying the quantitative trait loci (QTL) distribution across the Hamilton Eye Institute (HEI) Retina Database. Each point on the graph represents a single probe set. The x-axis plots the locations of the QTLs controlling the transcript expression. Positions are measured in mega bases (Mb) from chromosome 1 to chromosome X (1–2600 Mb). The y-axis plots the chromosomal location of each of the transcripts. The significant levels of individual QTLs are color-coded. The low genome wide p-value or high likelihood ratio statistic (LRS) are represented by red, the intermediate p-value/LRS values are green, and the high genome-wide p-value/low LRS values are presented in blue. The diagonal (red) band plots the large number of highly significant cis-acting QTLs and the vertical bands represent major trans-acting QTLs that co-regulate large numbers of downstream transcripts. These data were plotted from the HEI Retina Database with a false discovery rate of 0.2.

The HEI Retina Database presents the retinal transcriptome profiles of 75 BXD RI strains in a highly interactive website, GeneNetwork. The analytical tools within GeneNetwork allow for the a) systematic interrogation of the data set by identifying genetic variability across the BXD RI strains, b) constructing genetic networks controlling the development of the mouse retina, and c) defining the genomic loci underlying complex traits in the retina. To define genetic networks, quantitative trait analysis within GeneNetwork is used to reveal quantitative trait loci (QTLs) that modulate gene expression levels. Within the HEI Retina Database, there are a total of 11,141 probes with significant QTLs; 6,711 are trans-acting QTLs, while 4,078 QTLs are defined as cis-acting QTLs. The cis-acting QTLs are located at the same genomic locus as the gene of interest, while the trans-acting QTLs have genomic loci that modulate gene expression on a different location in the genome (for a complete discussion of trans-acting QTLs and cis-acting QTLs see [13]). The significant QTLs have a likelihood ratio statistic (LRS) over 15 (defined by GeneNetwork), which is the measurement of the linkage between the differences in mRNA expression and the differences in a particular DNA sequence. One way to visualize the total number of QTLs in the HEI Retina Database is to plot a genome-wide graph of all the QTLs, as shown in Figure 2. The graph displays the location of the best QTL for each probe set plotted against the corresponding location of the gene. The plot of the chromosomal location of each transcript with the corresponding position of the transcript’s largest QTL reveals the structure of the interactions of the transcripts within the BXD RI retina. The diagonal (red) band in Figure 2 represents the cis-acting QTLs, which are responsible for initiating the variations in gene expression. Generally, the cis-acting QTLs tend to have high LRS scores (>25). QTLs plotted away from this diagonal band represent the trans-acting QTLs and are most likely modulated by a cis-acting QTL at that genomic locus. On average, the LRS scores of the trans-acting QTLs tend to be lower than those of cis-acting QTLs. The co-regulated transcripts are represented as the vertical bands within the trans-acting QTLs. Thus, the retinal genome graph allows the visualization of the genetic sources of variation in the transcript expression of the retina at a global level.

### Cellular signature within the HEI Retina Database

**Figure 3:**
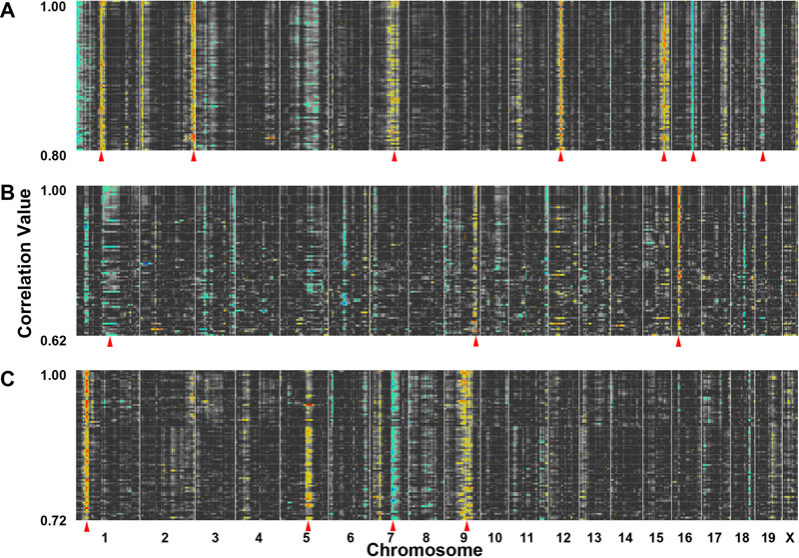
The cellular signatures of growth associated protein 43 (*Gap43*), retinal pigment epithelium-specific protein 65 kDa (*Rpe65*), and choline O-acetyltransferase (*Chat*) illustrated by quantitative trait loci (QTL) heat maps. The correlation matrices within GeneNetwork were used to simultaneous map and analyze the top 80 genes in the trait collections of A: *Gap43*, B: *Rpe65*, and C: *Chat*. The gene collections from *Gap43*, *Rpe65*, and *Chat* form unique signatures within the retina transcriptome. The numbers to the left denote the correlation range and at the bottom of the heat maps the chromosomal location is noted ranging from chromosome 1 on the left to chromosome X on the right. The banding pattern is displayed in yellow, red, green, and blue, which denote the locations of the genomic loci that modulate all of the genes in the network. The hues: yellow, red, green, and blue represent significant QTLs. The green (low LRS) to blue (high LRS) coloring represents transcripts whose expression is higher in the strains with a B haplotype (C57BL/6J) and the yellow (low LRS) to red (high LRS) coloring corresponds to the transcripts whose expression is higher in the strains with mutant D haplotype (DBA/2J). The red arrows indicate that some of the bands are more prominent. Running a QTL cluster map in GeneNetwork reveals a strong QTL signature of *Gap43*, forming a potential genetic network. Signature QTLs (highlighted with red arrows) for *Gap43* were found on chromosomes 1, 2, 7, 12, 15, 16, and 19. These are regions of the genome that contain loci modulating the expression of genes within the *Gap43* network (**A**). *Rpe65* forms signature QTLs on chromosomes 1, 9, and 16 (**B**) and *Chat* forms signature QTLs on chromosomes 1, 5, 7, and 9 (**C**).

The cellular signature of the genes that are uniquely expressed within specific retinal cell types can be identified within the HEI Retina Database; however, this does not mean that within a single cell type there is only one network. In fact, all cells will have multiple genetic networks that regulate specific cellular functions. A list of cell signature genes is presented in Table 1. For each of the cell signature genes listed in Table 1, GeneNetwork can be used to create a list of genes whose expression levels are highly correlated and form a unique signature of the expression pattern(s) within the specific cell type. For example, *Gap43* forms a retinal ganglion cell signature, and within the top 100 correlates of *Gap43* (see Appendix 2) we find genes such as G protein-coupled receptor 85 (*Gpr85*), Janus kinase1 (*Jak1*), heat shock protein 1 chaperonin 10 (*Hspe1*), neuron specific gene family member (*Nsg1*), translin (*Tsn*), beta helix-loop-helix domain containing, class B5 (*Bhlhb5*), cytochrome c, somatic (*Cycs*), and beclin 1, autophagy related (*Becn1*). The top 100 correlates of *Gap43* are a highly correlated list of genes with the 99th and 100th gene (male specific lethal-2 homolog1 [*Msl2l1*] and family with sequence similarity 108, member C [*Fam108c, RIKEN clone*]) having a correlation of r=0.79. The QTL heat map for *Gap43* (Figure 3A) reveals a unique set of genomic loci that regulate this list of genes while displaying a unique signature pattern of the genomic loci that modulate the genes expressed in retinal ganglion cells. The collection of genes forming these dense bands are termed signature QTLs [13]. Signature QTLs are genetic networks with a unique set of genomic loci that co-vary with the trait *Gap43*. Each band represents a region of the genome that modulates the collections of genes that are co-regulated within the specific cell type.

**Table 1:** Genetic signatures displayed in the Hamilton Eye Institute Retina Database

**Cell signatures**	**Signature genes**
Retinal Ganglion Cells	*Chrna6, Nrn1, Gap43, Thy1*
Amacrine Cells, Cholinergic Starburst	*Chat*
Amacrine Cells, GABA-ergic	*Gad1, Gad2*
Amacrine Cells, A17 Type	*Slc6a4*
Amacrine Cells, AII Rod Type	*Dab1, Gria3*
Amacrine Cells, Glycinergic	*Slc6a9*
Bipolar Cells	*Vsx2, Grm2*
OFF Cone Bipolar Cells	*Tacr3, Vsx1, Glrb*
ON Cone Bipolar Cells	*Gja7*
Rod Photoreceptors	*Rho*
Müller Glial Cells	*Slc1a3, Col9a1, Fzd5, Gpm6a, Mgll, Trim2, Rax, Qrsl2*
Retina Pigment Epithelium	*Rpe65, Rdh5, Rgr, Ttr*
Astrocytes	*Gfap*
Vasculature System	*Edg3, Efnb2, Nr2f2, Pecam1, Vcam1*
Circadian Rhythm	*Arntl, Clock, Per1*

Eleven different retinal cell type signatures including retinal ganglion cells, amacrine cells (5 types), bipolar cells (3 types), rod photoreceptors, muller glial cells, retina pigment epithelium, astrocytes, cell types involved in the vasculature system, and cells types involved in circadian rhythm are denoted in the left column and the genes associated with each cellular signature are displayed in the right column.

Within the *Gap43* network, signature QTLs are found on chromosomes 1, 2, 7, 12, 15, 16, and 19, as indicated by the red arrows in Figure 3A. This banding pattern is different from the signatures of other cell types within the retina, such as retinal pigment epithelial cells (*Rpe65*) and amacrine cells (*Chat*; Figure 3B,C). The *Rpe65* QTL heat map illustrates signature bands on chromosomes 1, 9, and 16 (Figure 3B), while the *Chat* QTL heat map presents signature bands on chromosomes 1, 5, 7, and 9 (Figure 3C). These cell-specific QTL signatures reflect the upstream modulators for the genes that are uniquely expressed within each specific retinal cell type. Furthermore, the QTL signatures reflect the functionally relevant genetic networks within the specific cell type.

### Examples of functional networks within the mouse retina

**Figure 4:**
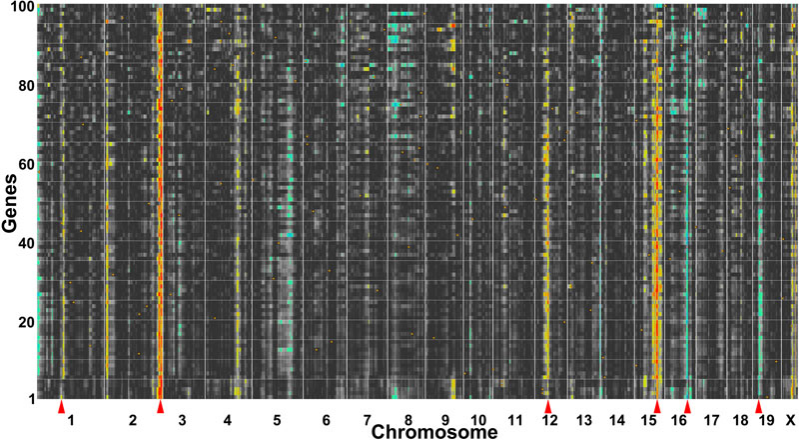
Quantitative trait loci (QTL) Heat Map of the top 100 correlates of phosphatase tensin homolog (*Pten*). The numbers to the left denote the top 100 genes that correlate with *Pten* and the chromosomal location is noted at the bottom of the heat map ranging from chromosome 1 on the left to chromosome X on the right. The banding pattern is displayed in yellow, red, green, and blue, which denote the locations of the genomic loci that modulate all of the genes in the network. The green (low likelihood ratio statistic; LRS) to blue (high LRS) coloring represents transcripts whose expression is higher in the strains with a B haplotype (C57BL/6J) and the yellow (low LRS) to red (high LRS) coloring corresponds to the transcripts whose expression is higher in the strains with mutant D haplotype (DBA/2J). The red arrows indicate the most prominent bands with significant QTLs and portray the signature bands of the *Pten* network chromosomes 1, 2, 12, 15, 16, and 19.

Our initial analysis displayed the *Gap43* signature as a cohort of transcripts co-regulated by the signature QTLs in the retinal ganglion cells. *Gap43* is of particular interest to our laboratory because of the *Gap43* association with growing axons and abortive regeneration in the retina [28,29]. When we examined the list of genes that correlate with the expression pattern of *Gap43* across the BXD RI strain set, *Pten* was discovered to have a relatively high correlation of 0.63 and has a similar expression pattern across the BXD RI strains. The high correlation is interesting because both genes, *Gap43* and *Pten*, are expressed in the retinal ganglion cells and *Pten* has been reported to be involved in axonal regeneration [30,31]. We hypothesize that *Pten* may share similar modulating QTLs to *Gap43*. Additionally, *Gap43* and *Pten* may participate within one molecular pathway in the retinal ganglion cells. Similar with the *Gap43* signature, the *Pten* signature displays QTLs on chromosomes 1, 2, 12, 15, 16, and 19 (Figure 4), which suggests that *Gap43* is co-regulated with *Pten* within the same genetic network of the retinal ganglion cells. This network shares a series of signature QTLs that may modulate the response of the ganglion cells to axonal injury: either abortive regeneration [32] or potentially axonal regeneration [30,31]. With further investigation of this network, it may be possible to define genes that act as modulators of both *Gap43* and *Pten*. For example, when the top 2,000 correlates of *Gap43* and *Pten* are compared (see Appendix 3), 1,109 genes are found to be in common, which indicates that *Gap43* and *Pten* participate within a single genetic network.

### Genes within the *Gap43/Pten* network

While analyzing the genetic networks of *Pten* and *Gap43*, several genes were discovered to have high correlations (0.5–1.0) with both *Pten* and *Gap43*. *Id2, Sox2, Pax6, Zbed4, Pcna*, and *Neurod1* were found in the top 800 correlates of both *Pten* and *Gap43*. Collectively and individually, these genes are known to be involved in both mouse and human retinal development. When the top 100 correlates for *Pax6, Sox2, Pten, Gap43, Id2, Neurod1, Zbed4*, and *Pcna* are mapped, the patterns are almost identical with two bands on chromosome 1, two bands on chromosome 2, one band on chromosome 12, one band on chromosome 15, one band on chromosome 16, and one band on chromosome 19. This indicates that these genes share common signature QTLs, suggesting that they are all part of the same genetic signature network.

**Figure 5:**
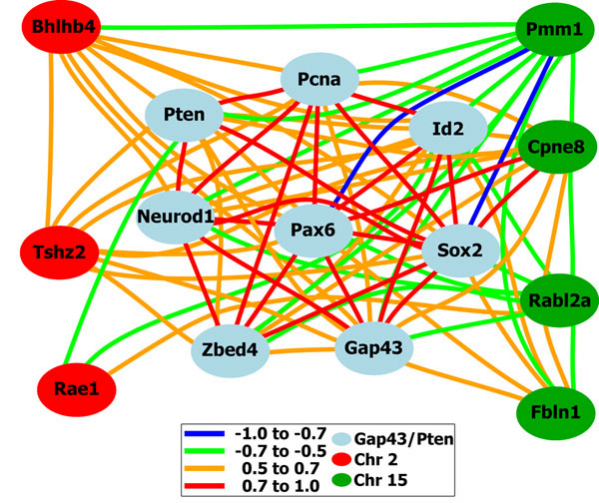
A network graph representing the growth associated protein43/ phosphatase tensin homolog (*Gap43/Pten*) network and the potential components for upstream modulation of the *Gap43/Pten* network. This network graph produces a relational *Gap43/Pten* network shown in blue. The colored lines represent the direct correlative relationship and the strength of the correlation is −1.0 to 1.0. The genes denoted in red: Basic helix loop helix beta4 (*Bhlhb4*), teashirt zinc finger homeobox 2 (*Tshz2*), and RNA export protein1 homolog (*Rae1*) are candidates from chromosome 2, cis-acting QTLs between 169 Mb and 182 Mb. The genes illustrated in green are the candidate genes located on chromosome 15 between 82 Mb and 92 Mb; phosphomannomutase1 (*Pmm1*), copine VIII (*Cpne8*), RAB, member of RAS oncogene family-like 2A (*Rabl2a*), and fibulin 1 (*Fbln1*).

A correlative network graph and correlation matrices are additional ways to view this group of genes that form the core of the *Gap43/Pten* network. Using the tools available within GeneNetwork, a network graph was computed for the *Gap43/Pten* network. The eight genes form the core of the *Gap43/Pten* network (light blue) are shown in Figure 5. All of these genes possess expression levels that highly correlate across the BXD RI strains (r=0.5–1.0). The correlations between each of these genes are color coded in the network graph illustrated in Figure 5 and presented in Table 2. We find that the average range of correlation is between 0.54 and 0.95. The lowest correlation is between *Id2* and *Zbed4,* with a value of 0.54, while the highest correlation (0.95) exists between *Sox2* and *Pax6*.

**Table 2:** Correlation matrix that displays the highly correlative expression levels of *Sox2, Pten, Gap43, Id2, Neurod1, Zbed4*, and *Pcna*.

**Gene symbol**	***Neurod1***	***Pax6***	***Pcna***	***Sox2***	***Id2***	***Gap43***	***Pten***	***Zbed4***
*Neurod1*	1	0.85	0.84	0.81	0.63	0.75	0.7	0.93
*Pax6*		1	0.88	0.95	0.73	0.68	0.63	0.84
*Pcna*			1	0.86	0.71	0.63	0.74	0.78
*Sox2*				1	0.76	0.68	0.65	0.8
*Id2*					1	0.64	0.67	0.54
*Gap43*						1	0.58	0.7
*Pten*							1	0.64
*Zbed4*								1

Correlation matrix that displays the highly correlative expression levels of the growth associated protein43/ phosphatase tensin homolog (*Gap43/Pten*) network including: beta loop helix beta transcription factors (sex determining region Y-box 2 [*Sox2*] and paired box gene 6 [*Pax6*]), a neuron specific transcription factor for neuronal development (neurogenic differentiation 1 [*Neurod1*]), genes involved in DNA binding (proliferating cell nuclear antigen [*Pcna*] and zinc finger, BED-type containing 4 [*Zbed4*]), and an inhibitor of DNA binding (inhibitor of DNA binding 2, dominant negative helix–loop–helix protein [Id2]). The gene symbols are located in the first column, and the first row of the table illustrates the corresponding levels of correlative expression (0.54–1.0).

To identify the intersecting sets of traits that correlate with *Pten, Neurod1, Sox2, Pax6, Id2, Pcna, Zbed4*, and *Gap43,* we performed a correlation comparison using the Pearson-correlation method with a threshold of 0.5. A correlation comparison aids in the determination of shared genetic correlations among groups of traits by correlating them with all the records from the HEI Retina Database. The results indicated that 1,287 sets of traits intersect with *Pax6, Sox2, Pten, Gap43, Neurod1, Pcna, Id2*, and *Zbed4*. By increasing the threshold we can focus on a more concentrated number of genes that are shared among the signature network. When the threshold value is set to 0.7, 88 genes were found to share genetic correlations among the *Gap43/Pten* network within the retina (see Appendix 4). These genes are tightly correlated forming a genetic network that may be involved with axonal growth and abortive regeneration.

To determine whether this network has a causal relationship(s), a partial correlation was performed between the genes of the *Gap43/Pten* network. A partial correlation is a correlation between two variables that remain after controlling for one or more other variables [33]. With this analysis networks can be interrogated by selecting a primary gene, a target gene, and control genes. If there is a significant difference, one can infer that the controlled variables have little effect and may not influence the variables or may not be a part of the model. It was determined that not all genes had a high correlation, indicating that all of the genes do not represent or share causal relationships. Two examples are as follows: *Zbed4* was chosen as the primary gene and *Id2* was selected as the control; the change in the Δ value for the target *Neurod1* was less than 0.05. When *Sox2* was selected as the control, *Pten* was selected as the primary gene and *Pax6* was listed as a target; the Δ value yielded 0.05. If the partial correlation exhibits a significant change, then there is a causal link between the variables and the change is dependent on the controlled variable. Numerous significant changes or causal relationships were found between the eight genes, with the majority of the genes having causal correlations above 0.8. Table 3 displays a few examples of the partial correlations that are believed to be of functional significance; however, they require further biologic testing.

**Table 3:** A sample table from a partial correlation table displaying examples of genes that have causal relationships by selecting a primary trait, control trait, and a target trait.

**Primary**	**Control**	**Target**	**Delta value**
*Pax6*	*Sox2/Pten*	*Id2*	0.8
*Pax6*	*Pcna/Zbed4*	*Neurod1*	0.94
*Sox2*	*Pcna/Zbed4*	*Neurod1*	0.97
*Sox2*	*Pax6/Pten*	*Neurod1*	0.98
*Sox2*	*Pax6/Pten*	*Zbed4*	0.92
*Zbed4*	*Pax6*	*Sox2*	0.87
*Neurod1*	*Pax6*	*Sox2*	0.82
*Id2*	*Sox2/Gap43*	*Pax6*	0.86

A sample of the partial correlation table displaying examples of the genes that have causal relationships within the growth associated protein 43/phosphatase tensin homolog (*Gap43/Pten*) network. Significant changes or causal relationships were identified between the genes in the *Gap43/Pten* network. The genes defined as having causal correlations display a value of 0.8 or above. The columns represent the selection of a primary trait (gene), control trait (gene), target trait (gene), and the significant change or delta value observed.

### Identifying candidate genes within the *Gap43/Pten* network

The HEI Retina Database and the tools available on GeneNetwork can aid in identifying potential candidate genes that are upstream modulators of a trans-acting QTL or signature trans-acting band. In order for a gene to be considered as an upstream modifier candidate of a genetic network, it must meet several minimal requirements. The first requirement is that the gene must be at the genomic location of the trans-acting band. If the gene is not at that genomic locus, then the gene could not be considered a candidate. Second, the expression of the gene should be highly variable across the BXD RI strain set. The variability allows the gene to be mapped and differentially influence gene expression. The third requirement is that the gene must have a significant LRS score (>17). An LRS score greater than 17 has a significant linkage (p value ≤0.05) and indicates that the gene can affect the phenotypic transcript expression. The expression level should be above the mean expression level of all the transcripts on the microarray (in GeneNetwork this is a value of 8 or higher). Additionally, there should be a high correlation (>0.5) between the gene and the network, indicating the theoretical/biologic relevance. Finally, an ideal candidate would have a literature connection between the candidate genes and the network. All of these criteria can be monitored using the tools and links within GeneNetwork (for a description of the use of these tools see [25]). For the purpose of illustration, we identified the candidate genes for the chromosome 2 and chromosome 15 trans-acting bands in the *Gap43/Pten* network.

### Candidate genes on chromosome 2

To identify the candidate genes that modulate the *Gap43/Pten* network, a search was performed for all genes with cis-acting QTLs having an LRS between 15 and 2,000, using a 20-Mb exclusion buffer, and a mean between 7.5 and 60 on chromosome 2 between 169 Mb and 182 Mb (distal band on chromosome 2). Within this region of the genome, GeneNetwork returns 18 records in which 16 genes are represented by 18 probes on the Illumina microarray (Table 4). The 13-Mb region on chromosome 2 contains 36 genomic markers with an average of less than 0.5 Mb between each marker, creating an accurate genomic map. When we examine the 16 genes present in this region, there are genomic markers separating each of the candidate genes, which rules out the possibility of linkage disequilibrium by genomic mapping. To narrow the candidate genes, we eliminated the four RIKEN clones, removed duplicate probe sets for teashirt zinc finger homeobox 2 (*Tshz2*), and deleted the genes that had a LRS<17 (RP23–330D3.5 and ADP-ridbosylation factor related protein 1 [*Arfrp1*]). Additionally, the genes that have an expression value less than 8 were also subtracted from the list (docking protein5 [*Dok5*], cadherin 4 [*Cdh4*], and potassium voltage-gated channel, subfamily Q, member 2 [*Kcnq2*]). The remaining seven candidate genes defined by this approach in the chromosome 2 trans-acting band are: *Tshz2,* RNA export 1 homolog (*Rae1*), opioid growth factor receptor (*Ogfr*), basic helix-loop-helix domain containing, class B4 (*Bhlhb4*), regulator of telomere elongation helicase1 (*Rtel1*), heat shock protein 40 kDa (*Hsp40 or DnaJ*) homolog, subfamily C, member 5 (*Dnajc5*), and transcription elongation factor A (SII), 2 (*Tcea2*).

**Table 4:** Genes displaying cis-acting QTLs on Chr 2 between 169 and 182 Mb.

**ID**	**Symbol**	**Description**	**ProbeSetID / RecordID**	**Probe target**	**Ch**	**Mb**	**UniGene ID**	**QTL Chr**	**QTL Mb**	**Locus at Peak**	**Max LRS**	**Mean expression**
1	Tshz2	teashirt zinc finger family member 2	ILMN_1246021	intron 2	2	169.6	Mm.315789	2	169.58	rs13476910	17.67	7.75
2	Tshz2	teashirt zinc finger family member 2	ILMN_1256408	exon 4	2	169.71	Mm.315789	2	168.79	rs6160839	17.9	8.01
3	Tshz2	teashirt zinc finger family member 2	ILMN_1243934	putative intergenic	2	169.74	Mm.315789	2	168.79	rs6160839	62.16	8.46
4	Dok5	docking protein 5	ILMN_2615070	exon 8	2	170.7	Mm.41633	2	173.43	rs3664044	17.35	7.95
5	Rae1	RAE1 RNA export 1	ILMN_2713130	3′ UTR	2	172.84	Mm.4113	2	172.56	rs3668691	45.41	10.87
6	RP23–330D3.5	novel KRAB box and zinc finger, C2H2 type domain containing protein	ILMN_2536053	intron 4	2	174.89	Mm.452080	2	172.56	rs3668691	15.55	8.14
7	Cdh4	cadherin 4	ILMN_1255939	exon 16	2	179.63	Mm.382094	2	179.26	rs6187766	30.81	7.88
8	AW120700		ILMN_1260036	putative intergenic	2	179.63		2	179.26	rs6187766	48.19	11.07
9	Ogfr	opioid growth factor receptor	ILMN_1250450	3′ UTR	2	180.33	Mm.250418	2	180.83	rs6305540	34.82	11.04
10	Bhlhb4	basic helix–loop–helix domain containing, class B4	ILMN_1227215	distal 3′ UTR	2	180.51	Mm.134062	2	179.7	rs3703298	31.4	8.44
11	Kcnq2	potassium voltage-gated channel, subfamily Q, member 2	ILMN_1241017	intron 8	2	180.84	Mm.40615	2	180.83	rs6305540	76.68	7.73
12	6330503H08Rik		ILMN_2564036	putative intergenic	2	180.87		2	180.83	rs6305540	23.9	8.18
13	2700038C09Rik	RIKEN cDNA 2700038C09 gene	ILMN_2590015	5′ UTR	2	180.92	Mm.213943	2	180.83	rs6305540	16.9	9.73
14	Rtel1	regulator of telomere elongation helicase 1	ILMN_2641946	3′ UTR	2	181.09	Mm.11333	2	180.83	rs6305540	68.62	8.6
15	Arfrp1	ADP-ribosylation factor related protein 1	ILMN_2631745	exon 6 (high cisQTL in BXD Striatum Illumina with D high allele, no SNP)	2	181.1	Mm.87720	2	180.83	rs6305540	16.15	11.23
16	Dnajc5	DnaJ (Hsp40) homolog, subfamily C, member 5	ILMN_2501026	putative intergenic	2	181.29	Mm.140761	2	180.83	rs6305540	117.47	14.35
17	Tcea2	transcription elongation factor A (SII), 2	ILMN_2777609	3′ UTR	2	181.42	Mm.24245	2	180.83	rs6305540	70.53	10.58
18	2810410D24Rik	RIKEN cDNA 2810410D24 gene	ILMN_2493144	putative intergenic	2	181.6	Mm.432139	2	179.7	rs3703298	22.87	7.77

Eighteen records were observed to have cis-acting quantitative trait loci (QTL) on chromosome 2 between 169 and 182 Mb. Eighteen gene symbols are displayed in the table as well as the description of the gene, Probe Set Identification, probe target, chromosomal location, Megabase, UniGene identification, chromosome in which QTL is located, locus at peak, the maximum likelihood ratio statistic (LRS), and mean expression.

### Candidate genes on chromosome 15

A similar analysis was conducted to determine the candidate genes within the trans-acting signature band found on chromosome 15. Using the advanced search features in GeneNetwork, we performed a search of the cis QTLs that have an LRS between 15 and 2,000, using a 20-Mb exclusion buffer, a mean between 7.5 and 60, and with target genes on chromosome 15 between 82 and 92 Mb. Within the 10-Mb region of chromosome 15, there are 42 genomic markers, which provide a high mapping density. The search returns 20 records displaying 16 genes (Table 5). To narrow our search, the RIKEN clone and the duplicate probe sets for phosphomannomutase 1 (*Pmm1*), X-ray repair complementing defective repair in Chinese hamster cells6 (*Xrcc6*), malonyl CoA-acyl carrier protein acyltransferase, mitochondria (*Mcat*), and RAB, member of RAS oncogene family-like 2A (*Rabl2a*) were removed from the list in Table 5. Serine hydrolase-like (*Serh1*) and fibulin1 (*Fbln1*) can be deleted from the selected genes because they do not display a mean expression value of 8.0 and aconitase 2, mitochondrial (*Aco2*). can be eliminated from the list due to the LRS being below 17. *Zbed4*, one of the genes displayed within the *Gap43/Pten* network, has a cis-QTL on chromosome 15 at 87.3 Mb and its expression is highly correlated with the other genes in the *Gap43/Pten* network, as shown in Figure 5. As a result, this suggests that *Zbed4* would make an excellent candidate gene for the *Gap43/Pten* network; however, we have left *Zbed4* out of our candidate gene analysis because the *Zbed4* analysis was previously performed with the *Gap43/Pten* network. Thus, it was established that 11 genes met the search criteria: PHD finger protein5A (*Phf5a*), polymerase (RNA) III (DNA directed) polypeptide H (*Polr3h*), phosphomannomutase 1 (*Pmm1*), X-ray repair complementing defective repair in Chinese hamster cells6 (*Xrcc6*), malonyl CoA-acyl carrier protein acyltransferase, mitochondria (*Mcat*), PHD finger protein 21B (*Phf21b*), TBC1 domain family, member 22a (*Tbc1d22a*), arylsulfatase A (*Arsa*), RAB, member of RAS oncogen gamily-like 2A (*Rabl2a*), copine VIII (*Cpne8*), and kinesin family member 21A (Kif21a). Additionally, there were two genes, peroxisome proliferator-activated receptor (*Ppara*) and fibulin 1 (*Fbln1*), which appeared to be good candidate genes; however, these genes did not meet the criteria initially used in the search. *Ppara* has a strong cis-acting QTL with an LRS score of 59.7; however, the expression level of *Ppara* is two-fold below the mean expression on the microarrays. Even though *Ppara* has a lower expression level than our initial criteria, it was included in the candidate gene list due to its strong cis-acting QTL. *Fbln1* was included in the candidate gene list for similar reasons; it has as strong cis-QTL (LRS=24) and was just below (7.6) the expression cut-off criterion of 8.0.

**Table 5:** Genes displaying cis-acting QTLs on Chr 15 between 82 and 92 Mb.

**ID**	**Symbol**	**Description**	**ProbeSetID / RecordID**	**Probe Target**	**Chr**	**Mb**	**UniGene ID**	**QTL Chr**	**QTL Mb**	**Locus at Peak**	**Max LRS**	**Mean Expression**
1	Phf5a	PHD finger protein 5A	ILMN_1251761	3′ UTR	15	81.7	Mm.271715	15	82.3	D15Mit189	32.6	13.6
2	Aco2	aconitase 2, mitochondrial	ILMN_1235786	exon 19	15	81.7	Mm.154581	15	79.7	rs4230879	15.6	12.4
3	Polr3h	polymerase (RNA) III (DNA directed) polypeptide H	ILMN_1218993	3′ UTR	15	81.7	Mm.215115	15	79.5	rs4230869	21	10.9
4	Pmm1	phosphomannomutase 1	ILMN_2611027	exon 7	15	81.8	Mm.18939	15	82.3	D15Mit189	30.4	12.3
5	Pmm1	phosphomannomutase 1	ILMN_1223710	5′ UTR	15	81.8	Mm.18939	15	83.9	rs13482681	16.8	10.3
6	Xrcc6	X-ray repair complementing defective repair in Chinese hamster cells 6	ILMN_1247814	exon 13	15	81.9	Mm.288809	15	79.7	rs4230879	18.6	9.6
7	Xrcc6	X-ray repair complementing defective repair in Chinese hamster cells 6	ILMN_2862619	3′ UTR	15	81.9	Mm.288809	15	79.5	rs4230869	42.6	12
8	Serhl	serine hydrolase-like	ILMN_2614351	exon 11	15	82.9	Mm.379160	15	82.9	rs13482677	130.8	7.7
9	Mcat	malonyl CoA:ACP acyltransferase (mitochondrial)	ILMN_2619455	3′ UTR	15	83.4	Mm.37560	15	82.9	rs13482677	97.8	10.6
10	Mcat	malonyl CoA:ACP acyltransferase (mitochondrial)	ILMN_2927635	exon 3	15	83.4	Mm.37560	15	83.9	rs13482681	76	7.6
11	Phf21b	PHD finger protein 21B	ILMN_1229266	3′ UTR	15	84.6	Mm.101022	15	87.9	rs3707587	28.9	11.6
12	Fbln1	fibulin 1	ILMN_2602770	3′ UTR	15	85.1	Mm.297992	15	82.9	rs13482677	24.1	7.6
13	2210021J22Rik	RIKEN cDNA 2210021J22 gene	ILMN_2781118	exon 4	15	85.6	Mm.33706	15	83.9	rs13482681	36.4	8.1
14	Tbc1d22a	TBC1 domain family, member 22a	ILMN_2767791	3′ UTR	15	86.3	Mm.28904	15	85.5	rs13482686	30.3	11.4
15	Zbed4	zinc finger, BED domain containing 4	ILMN_2418222	3′ UTR	15	88.6	Mm.397231	15	87.3	rs6405854	17.6	9.4
16	Arsa	arylsulfatase A	ILMN_1225552	3′ UTR	15	89.3	Mm.620	15	88.3	rs13482695	42	10
17	Rabl2a	RAB, member of RAS oncogene family-like 2A	ILMN_2593578	3′ UTR	15	89.4	Mm.23258	15	88.3	rs13482695	36.6	12.2
18	Rabl2a	RAB, member of RAS oncogene family-like 2A	ILMN_2742942	exon 7	15	89.4	Mm.23258	15	89.4	rs4230970	69.9	7.6
19	Cpne8	copine VIII	ILMN_3151149	intron 2	15	90.5	Mm.290991	15	87.9	rs3707587	22.7	9.8
20	Kif21a	kinesin family member 21A	ILMN_2718716	exon 33	15	90.8	Mm.41379	15	89.4	rs4230970	25.1	12.3

Twenty records were observed to have cis-acting quantitative trait loci (QTL) on chromosome 15 between 82 and 92 Mb. Twenty gene symbols are displayed in the table as well as the description of the gene, probe set identification, probe target, chromosomal location, megabase location, uniGene identification, chromosome in which QTL is located, Locus at peak, the maximum likelihood ratio statistic (LRS), and mean expression.

### Using network correlation to prioritize candidate genes

Additional criteria can be used to narrow the list of candidate genes. Our initial analysis of the chromosome 2 locus revealed seven potential genes are upstream modulator candidates of the *Gap43/Pten* network and chromosome 15 has thirteen potential candidate genes. With such a large number of candidate genes (20 total candidate genes), it is difficult to begin to plan any biologic experiments to validate which gene is modulating the network. One approach is to identify genes that show similar expression(s) patterns as the other genes within the network that they are potentially modulating. Thus, the candidate genes were prioritized based upon the genes that are most likely to have a direct effect on the *Gap43/Pten* network. Within the GeneNetwork, the candidate genes for the *Gap43/Pten* network from chromosome 2 and chromosome 15 (Table 4 and Table 5) are added to a collection with the core of the *Gap43/Pten* network (*Gap43, Pten, Pcna, Id2, Neurod1, Zbed4, Pax6,* and *Sox2*). The network correlation program was processed, we selected only the candidate genes with a high correlation (0.5), and the most direct connection to the network genes (Figure 5). These criteria narrowed our search to seven genes (*Tshz2, Rae1, Bhlhb4, Rabl2a, Pmm1, Cpne8*, and *Fbln1*) that could be considered as potential candidates for modulating the *Gap43/Pten* network.

Within the distal signature band on chromosome 2, it was determined that *Tshz2, Rae1*, and *Bhlhb4* are located within the top 2,000 correlates for *Pten*. This correlation in the expression pattern across the BXD RI strain set is a strong indicator that these genes are potential candidates for an upstream modulator of the trans-acting band on chromosome 2. *Rae1* has a negative correlation with *Id2* (−0.53) and *Pten* (−0.54). *Bhlhb4* positively correlates with *Pcna* (0.53), *Neurod1* (0.5), *Sox2* (0.55), *Id2* (0.54), *Pten* (0.68), *Pax6* (0.5), and *Gap43* (0.56). *Tshz2* correlates with *Bhlhb4* (0.54), *Gap43* (0.52), *Id2* (0.53), *Pax6* (0.54), *Pcna* (0.55), *Sox2* (0.66), *Pten* (0.54), and *Zbed4* (0.51). Thus, it appears that on the chromosome 2 locus there are three potential candidate genes: *Rae1* (a potential negative modulator of the network), *Bhlhb4* (a putative positive modulator of the network), and *Tshz2* (putative positive modulator of the network).

The potential candidates for chromosome 15 were *Rabl2a, Pmm1, Cpne8*, and *Fbln1. Pmm1* is the only candidate gene located in the top 2,000 correlates of *Pten*; however, *Cpne8, Pmm1*, and *Rabl2a* were found in the top 2,000 correlates of *Gap43*. Through this analysis, both potential positive and negative modulators of the network were found as indicated below. *Rabl2a* has a negative correlation value of 0.5–0.7 with *Pax6* (−0.57), *Id2* (−0.62), *Fbln1* (−0.52), *Gap43* (−0.61), *Cpne8* (−0.68), and *Zbed4* (−0.51). *Pmm1* also has a negative correlation with several genes in the network, such as *Pax6* (−0.68), *Pcna* (−0.7), *Sox2* (−0.72), *Zbed4* (−0.57), *Neurod1* (−0.56), *Gap43* (−0.59), *Cpne8* (−0.57), *Id2* (−0.62), and *Fbln1* (−0.57). *Cpne8* displays a positive correlation with *Pax6* (0.71), *Pcna* (0.64), *Sox2* (0.77), *Zbed4* (0.62), *Id2* (0.63), *Fbln1* (0.57), *Gap43* (0.65), *Pten* (0.5), and *Neurod1* (0.63); however, *Cpne8* negatively correlates with *Rabl2a* (−0.65) and *Pmm1* (−0.62). *Fbln1* has positive correlations with *Pax6* (0.6), *Sox2* (0.64), *Id2* (0.68), *Cpne8* (0.62), and *Gap43* (0.51); however, *Fbln1* also has negative correlations with *Pmm1* (−0.56) and *Rabl2a* (−0.59).

Another approach to minimize and prioritize the candidate genes is to perform a literature search. Although sparse, we unveiled literature correlations for at least four of the candidate genes. *Bhlhb4* is a transcription factor that is required for the rod bipolar cell maturation [34]. It is well-known that the bipolar cells receive input from the cones and many feed into the retinal ganglion cells [35]. *PMM1* has been associated with a degenerative disorder, CDG1 (carbohydrate-deficient glycoprotein deficient syndrome type I), which can present ocular degeneration symptoms [36-38]. *FBLN1* was recently discovered as a downstream target of SP1 transcription factor (*SP1*) in a manuscript regarding the network analysis of human glaucoma [39]. *RABl2A* is ubiquitously expressed in humans [40] and has been associated with F379, which is a retina-specific transcript [41]. Thus, the literature search implicates that four of the seven candidate genes, *Bhlhb4, Fbln1, Rabl2a,* and *Pmm1*, would be valid components of *Gap43/Pten* network.

## Discussion

There is a tremendous need to develop interactive tissue databases to organize, sort, and mine the vast amount of genomic data. This is especially true if we are to define the genetic basis of complex traits and diseases. Over the years many approaches and technologies have been created to identify genes that are expressed in the retina, such as expressed sequence tag information [6,42-47], sequencing cDNA libraries generated by conventional methods or normalization techniques [48-55], hybridization to gene arrays of various formats [56,57], and serial analysis of gene expression [46,47,58,59].

Many of these approaches are now available as databases, and we have summarized them in Table 6. In 2009, the National Eye Institute held a workshop to help identify the gaps, needs, and opportunities in ophthalmic genetics. This included ophthalmic disease conditions, biologic systems, and approach/methodologies. A few concerns were identifying the major genes and the modifiable risk factors that cause glaucoma, identification of the modifier genes for retinal degeneration, appropriate animal models to study complex disease, and approaches for statistical genetics. The HEI Retina Database, which combines 75 BXD RI strain sets with systems biology, is a unique and powerful approach that will help to address some of these questions. The HEI Retina Database is an interactive website within GeneNetwork that provides a transcriptome-wide analysis of the retina, thus enabling the study of genetic networks present within the retina.

**Table 6:** Summary of retina databases currently available.

**Databases**	**Contents**
National Eye Institute: NEI Bank Project	Transcriptome profiling for different tissues of the eye for a variety of different species including mouse and human
Mouse Retina Serial Analysis of Gene Expression (SAGE)	Gene expression data from the embryonic and postnatal mouse retina.
Scripps Friedlander Gene Expression	Expression of retinal transcripts for the mouse retina during eight different postnatal stages.
Retinal Information Network	Mapped loci and cloned genes associated with inherited retinal disease.
Retinal International Website	Series of databases listing: retinal diseases, mutations affecting the eye, current animal models, and known proteins involved in retinal disease.
GENSTAT: Retina Project	Cell specific labeling in the retina using BAC transgenic mice.
Retina Central	Integrated platform for gene related information regarding the adult mammalian retina

The summary table includes the database names, followed by a brief summary of the contents of the database, and the website address associated with the retina databases.

The HEI Retina Database provides a powerful model to study the genetic covariance of the 75 BXD RI strains because it allows researchers to identify cellular signatures and genetic networks within the retina. Both the Hamilton Eye Institute Mouse Expression Database (HEIMED) and HEI Retina Database in conjunction with GeneNetwork enhance the power of the bioinformatic resources listed in Table 6 by providing unique sets of bioinformatic research tools. The HEI Retina Database characterizes the genetic networks regulating the complex structure of the retina, identifies the variation in retina phenotypes and disease susceptibility, and determines the gene variants that have expression patterns closely associated with higher order phenotypes. Thus, the HEI Retina Database provides a wealth of research possibilities, including: a) defining the molecular signatures within the retina, b) ascertaining the unique cell signatures for a specific cell type, c) identifying candidate genes for human disease, d) ability to understand genetic networks regulating tissue-specific gene expression, and e) determining the complex interactions of the genomic loci that underlie the complex structures of the retina. This approach may lead to additional studies further evaluating system genetics and complex trait analysis using the links within GeneNetwork such as UCSC Genome Browsers, Ensembl Genome Browser, PubMed, Entrez Gene, ABI panther, and WebGestalt.

One example of the discovery of genetic networks within the HEI Retina Database/GeneNetwork is the *Gap43/Pten* network. This example provides a unique genetic signature for retinal ganglion cells. The QTL heat map for *Gap43* led us to a unique cellular signature that is similar to *Pten*, which suggests that the two genes may participate in one molecular pathway in retinal ganglion cells. Additionally, the similarity suggests that *Gap43* co-regulates *Pten* with a collection of genes (*Pax6, Sox2, Id2, Neurod1, Zbed4*, and *Pcna*) that are strongly associated with this network. These genes possess the same genetic signature network as indicated by the common signature QTLs on chromosome 1, 2, 15, 16, and 19 as well as displaying high correlations between the genes (>0.5). Additional interrogation of the *Gap43/Pten* network indicated a causal link between some of the genes, as displayed in Table 2. GeneNetwork pinpointed the potential upstream modifier candidates for the *Gap43/Pten* network with implementation of stringent criteria (as discussed within the results section): a) the genomic location of the trans-acting band and gene of interest must co-exist, b) high variability in expression levels across the strain set, c) significant LRS scores (>17), d) mean expression above 8, e) high correlations between the gene of interest and the network (>0.5), as well as f) a literature correlation between the network and gene of interest. Using these criteria, seven candidates from chromosome 2 and thirteen candidates from chromosome 15 were selected as potential upstream modifiers of the *Gap43/Pten* network. Based upon the similar expression patterns, strong correlations (>0.5) in the expression patterns across the BXD RI upstream modulators, and a direct connection to the network genes (*Pax6, Sox2, Id2, Neurod1, Zbed4*, and *Pcna*), the search for candidates was narrowed to seven genes of interest (*Tshz2, Rae1, Bhlhb4, Rabl2a, Pmm1, Cpen8*, and *Fbln1*), as shown in Figure 5.

Throughout the literature, several studies have attempted to determine retinal signature genes and networks. Three recent publications indicate a direct link to our systems biology approach and the study of genetic networks in the retina [60-64]. Publications by Ivanov and colleagues [60] were of particular interest to our laboratory because they are identifying the retinal ganglion cell enriched genes that are preferentially expressed in the adult retinal ganglion cells. At least five candidate genes from the study of differential gene expression profiling of retinal ganglion cells [60] share similar QTL heat maps to the *Gap43/Pten* network: mitogen-activated protein kinase 1 (*Map2k1*), coagulation factor C homolog, cochlin (*Coch*), tyrosine 3-monooxygenase/tryptophan 5-monooxygenase activation protein, beta polypeptide (*Ywhab*), tyrosine 3-monooxygenase/tryptophan 5-monooxygenase activation protein, zeta polypeptide (*Ywhaz*), and plastin 3 (*Pls3*). The similarity between the QTL heat maps as well as the molecular functions of these genes (*Map2k1, Coch, Ywhab, Ywhaz*, and *Pls3*) suggests that they are likely to participate in the *Gap43/Pten* signature network [60-63]. Recent studies of the expression changes in retinal ganglion cells further indicate that *Ywhab* and *Ywhaz* [60-63] are strong candidates for the *Gap43/Pten* network. *Ywhab* and *Ywhaz* are of interest because their chromosomal location is associated with the *Gap43/Pten* network, chromosome 2 and chromosome 15, respectively, and they share 99% homology in the mouse and rat. Both *Ywab* and *Ywhaz* have been linked to the mitogenic signaling and cell-cycle machinery within the retina, as demonstrated by the genes in which they associate and interact. *Ywhaz’s* encoded protein is known to interact with CDC25B [65], C-RAF [66-70], AKT1 [71], P53 [72], and BCL2 [73]. *Ywhab’s* encoded protein is known to interact with RAF and BRAF [74,75], and CDC25A/B [65,76]. It is possible that the involvement of *Ywhab* and *Ywhaz* in the *Gap43/Pten* network may be to preserve the retinal ganglion cell homeostasis since retinal ganglion cells are one of the most metabolically active/energy demanding cell types and they depend upon the increased activity of prosurvival genes to sustain their normal function [77,78].

The future of this approach will extend to defining the cell intrinsic pathways that are responsible for susceptibility of retinal ganglion cell death and identifying networks that underlie the response of retinal injury. Additionally, this approach will help pinpoint the complex genetic networks that regulate inherited retinal diseases such as age related macular degeneration, glaucoma, and diabetic retinopathy. We anticipate that the interactive HEI Retinal Database and the databases listed in Table 6 will help to alleviate the major challenges in identifying and validating the genes that contribute to retinal disease phenotypes as well as the genetic and regulatory mechanisms that underlie the complex trait diseases of the retina.

## Appendix 1.

HEI Retina case IDs, including sample tube ID, strain, age, sex, and source of mice. To access the data, click or select the words “Appendix 1.” This will initiate the download of a compressed (pdf) archive that contains the file. Hamilton Eye Institute Retina Database case IDs, including sample tube ID, strain, age, sex, and source of mice.

## Appendix 2.

Top 100 gene correlates of growth associated protein 43 (*Gap43*). To access the data, click or select the words “Appendix 2.” This will initiate the download of a compressed (pdf) archive that contains the file.

## Appendix 3.

The genes shared in the top 2,000 correlates of growth associated protein 43 (*Gap43*) and phosphatase tensin homolog (*Pten*). To access the data, click or select the words “Appendix 3.” This will initiate the download of a compressed (pdf) archive that contains the file.

## Appendix 4.

List of genes that share genetic correlations among the growth associated protein 43/ phosphatase tensin homolog (*Gap43/Pten*) network within the retina. To access the data, click or select the words “Appendix 4.” This will initiate the download of a compressed (pdf) archive that contains the file.
